# Microbiology Biotechnology in China

**DOI:** 10.1111/1751-7915.13777

**Published:** 2021-02-20

**Authors:** Wei E. Huang, Chen Yang, Hui Wang, Lingquan Bai, Ning‐Yi Zhou

**Affiliations:** ^1^ Department of Engineering Science University of Oxford Parks Road Oxford OX1 3PJ UK; ^2^ Centre for Excellence in Molecular Plant Sciences, Shanghai Institute of Plant Physiology and Ecology Chinese Academy of Sciences Shanghai China; ^3^ Environmental School Tsinghua University Beijing China; ^4^ State Key Laboratory of Microbial Metabolism, School of Life Sciences and Biotechnology Shanghai Jiao Tong University Shanghai China

*A social demand plays a more stimulating role to promote advances in science and technology than 10 universities together*, a thinker in 19th Century has said. Microbial biotechnology has been significantly stimulated due to the social demand in China over the past decades. With rapid economic growth in China, the public and government demand on good environmental quality has requested microbial biotechnological solutions to large scale and complex contamination in air, water and soils. With recent advances on synthetic biology and microbial biotechnology, microbes have been found to be economically viable working horses to carbon economy and biomanufacturing.

China’s rapid economy growth has also stimulated environmental microbiology research in various areas such as agriculture, aquaculture, deep sea ecosystems, green bioenergies and bioremediation. Both government and research funding bodies in China strongly supported research on microbial biotechnology and provided a huge budget on the applications. As a result, Chinese advances in basic and applied microbiology and biotechnology are leading to significant improvements in agriculture, aquaculture, deep sea mining, bioenergies and biofuels, and the use of microbes to break down contaminants.

As one of the top journals in the field of biotechnology & applied microbiology, Microbial Biotechnology (MBT) is attracting an increasing number of key publications from the Chinese Academy of Sciences, research institutes and universities.

Here in this special issue of ‘*Advances in Microbial Biotechnology in China*’, Chinese scientists and their international collaborators explored broad topics including synthetic biology, metabolic engineering, new methods of genomics and biomarkers, biodegradation, fecal microbiota, synthetic community, industrial enzymes and directed evolution, interactions between bacteria and phage, fungus and plants, green biotechnology, biosynthesis of active molecules and new polyketides.

